# An Algorithmic Approach to Managing Parotid Duct Injury Following Buccal Fat Pad Removal

**DOI:** 10.1093/asjof/ojac032

**Published:** 2022-04-25

**Authors:** Jason M Weissler, Omar Mohamed, Joseph M Gryskiewicz, Karan Chopra

**Affiliations:** Division of Plastic Surgery, Department of Surgery, Mayo Clinic College of Medicine and Science, Rochester, MN, USA; Division of Plastic Surgery, Department of Surgery, Mayo Clinic College of Medicine and Science, Rochester, MN, USA

## Abstract

The principles of achieving an aesthetically pleasing and harmonious facial appearance are influenced by our evolving understanding of the three-dimensional topography of the face coupled with novel approaches to midface volumization and contouring. In parallel with the evolving landscape of facial aesthetic surgery, an increasing number of publications have emerged focusing on the role of intraoral buccal fat pad removal (BFPR) for the purposes of aesthetic midface contouring. The authors sought to emphasize an underreported and potentially preventable and untoward complication involving iatrogenic parotid duct injury following BFPR. The purpose of this publication is 3-fold: (1) to review the relevant anatomy and literature on intraoral BFPR, (2) to present a case example of this complication, and (3) to discuss treatment options as part of a proposed management algorithm. The authors detail the surgical indications for performing BFPR and review the relevant anatomic considerations. Complication prevention strategies are outlined, and details regarding avoidance of parotid duct injury are reviewed. The diagnostic criteria and clinical presentation of parotid duct injuries following BFPR are comprehensively outlined. The authors present a clinically actionable algorithm for managing a suspected or diagnosed parotid duct injury following BFPR. A clinical case example is discussed to highlight this complication, the diagnostic criteria, and the systematic and algorithmic approach to management. Intraoral BFPR is an incredibly valuable and powerful procedure that can improve facial contour in patients with buccal lipodystrophy or buccal fat pad pseudoherniation. The authors propose a proper diagnostic approach and treatment algorithm to manage this untoward complication.

**Level of Evidence: 5:**

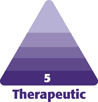

The principles of achieving an aesthetically pleasing and harmonious facial appearance are influenced by our evolving understanding of the three-dimensional facial topography coupled with sound approaches to midface volumization and contouring. Although the basic tenets of improving the appearance of the midface remain generally unchanged, more recently, an increasing number of surgeons have been incorporating procedures such as intraoral buccal lipectomy to address the fullness of the midface to accentuate a slimmer facial appearance.

While the intraoral approach to buccal fat pad removal (BFPR) was first described by Epstein in 1980 and later popularized by Stuzin et al and Matarasso,^1-3^ procedures to address midface fullness have recently experienced resurgence in the plastic surgery literature and have gained increased attention through social media platforms.^4-6^ In parallel with the evolving landscape of facial aesthetic surgery, an increasing number of publications have emerged focusing on the role of intraoral BFPR for the purposes of aesthetic midface contouring.^3,7-11^ Most of the available literature concentrates on the clinically relevant anatomic relationships of the buccal fat pad to the adjacent anatomic structures which are at risk of injury during resection, such as the parotid duct and buccal branches of the facial nerve. Furthermore, recent work by Rohrich et al raised concern regarding the lack of long-term data to support BFPR, while the procedure itself may potentially promote premature aging and midface distortion over time.^12^

With regard to the risk profile of this procedure as it pertains to iatrogenic parotid duct injury, when performed for facial slimming, this complication is rare and likely underreported.^13^ To date, the literature on iatrogenic injury to the parotid following facelift surgery or intraoral BFPR remains limited. As such, there remains a paucity of information regarding the incidence, diagnosis, and systematic approach to managing iatrogenic parotid duct injury after facial aesthetic procedures, specifically BFPR. Given the intimate anatomic relationship among the buccal fat pad, Stensen’s duct, and facial nerve branches, aesthetic surgeons who perform BFPR should have a thorough understanding of the relevant anatomy to mitigate inadvertent injury to surrounding structures. Surgeons should also be familiar with the clinical presentation, diagnostic considerations, and available treatment options to manage iatrogenic injury of the parotid duct following BFPR.

The purpose of this publication is 3-fold: (1) to review the relevant anatomy and literature on intraoral BFPR, (2) to present a case example of an iatrogenic parotid duct injury and its management, and (3) to discuss a systematic and algorithmic approach for management of this complication.

## BACKGROUND

Our understanding of what contributes to an aesthetically pleasing midface is founded on the following 5 components, as emphasized by Matarasso: a distinct transition between the anterior border of the parotid gland and cheek hollow, visibility of the posterior border of the nasolabial fold, a subtle submalar depression, prominent zygomatic eminences, and a well-defined mandibular angle.^3-5^ To effectively achieve the desired midface proportions based on these principles, there are a variety of surgical and minimally invasive modalities available to aesthetic surgeons, which through either volumetric addition or subtraction contribute to a well-balanced aesthetic appearance. One such technique that has become increasingly popularized through social media platforms is BFPR, which can be performed either in the operating room or in an office setting under local anesthesia.

For properly selected patients, the buccal fat pad can be removed either intraorally or extraorally during rhytidectomy. It can also be performed as a standalone procedure or in conjunction with other procedures, such as neuromodulators and fillers, contouring of the facial skeleton, and autologous fat transfer, for instance.^3^ Although intraoral BFPR alone is a simple and effective technique for contouring the midface with an acceptable safety profile, surgeons who perform these procedures should be cognizant of both the relevant anatomy and how the intimate relationship of certain critical structures may contribute to inadvertent iatrogenic injury, as well as the potential long-term ramifications on the effects of advanced facial aging which remain unknown.^12^

### Surgical Indications

Patient selection is paramount when considering performing BFPR. Treatment of the widened midface is dependent upon an assessment of the factors contributing to a wide midface.^3-5^ The procedure can be considered in most age groups to treat either buccal lipodystrophy or displacement of the buccal fat pad (pseudoherniation), both of which present with fullness within the cheek region. Ultimately, the goal of BFPR is to enhance the appearance of the midface by reducing cheek fullness while accentuating the malar eminences. Patients with malar hypoplasia would be poor candidates for this procedure because BFPR may further emphasize a hollowed-out cheek appearance and detract from aesthetic facial proportions.^2^

### Anatomic Considerations and Complication Prevention Strategies

Given the valuable clinical applications of the buccal fat pad in aesthetic surgery, much attention has been given to its anatomic description. Originally described by Heister and Bichat, the buccal fat pad is a triangular-shaped and tubular adipose structure closely associated with the muscles of mastication within the cheek.^1,2^ It carries both functional reconstructive and aesthetic clinical value as an anatomic structure capable of reconstructing oronasal fistulas and oncologic defects and contouring the midface for patients with mid/lower facial fullness.^14-16^

The buccal fat pad is unique in the sense that unlike other fat compartments in the face and body, the buccal fat maintains a constant volume throughout life. It is often a common culprit for the persistence of midface fullness despite weight loss efforts, as it has also been found to be hormonally insensitive and unresponsive to weight fluctuations.^4,6^ Furthermore, possible contributors to the development of midface fullness are displacement of buccal fat, herniation of buccal fat secondary to weakness in the anterior Superficial musculoaponeurotic system (SMAS), or pseudoherniation of the fat pad secondary to weakening of investing fascia.^2-6,12,15^

Anatomic descriptions demonstrate that the buccal fat pad consists of a main body positioned centrally with 4 extensions: buccal, pterygoid, superficial, and deep temporal.^2,12^ The buccal extension is the most superficial segment and is the portion that contributes to cheek fullness. When accessing the buccal fat pad intraorally, the goal is to safely remove the main body and the buccal segment, which together contribute to approximately half of the total fat volume within the buccal fat pad. It is also important to note that the buccal fat pad is unique in that it is found in both the superficial and deep fat compartments.^12,16^

Perhaps the most important consideration is to respect the anatomical relationships between the buccal branch of the facial nerve and the parotid duct. Although a thorough discussion of facial nerve anatomy in relation to the buccal fat pad is beyond the scope of this work, knowledge of the variability of the nerve in relation to the parotid duct and buccal fat is critical in preventing unintentional injury. For these reasons, this procedure is not without risk, and patients must be counseled on the possible risks of injury to these structures.

With regard to the anatomic relationship between the parotid duct and the buccal fat pad, most of the anatomic descriptions have been largely based on cadaveric facial dissections.^2,12,16^ Most studies emphasize the vulnerability of the parotid duct to inadvertent injury given the close proximity of the course of the parotid duct to the buccal fat pad. Familiarity with the parotid duct’s course and its associated surface landmarks such as the line extending from the tragus to the midportion of the upper lip can further mitigate the risk of inadvertent injury when accessing the buccal fat pad, specifically through a facelift approach. Although more applicable to extraoral approaches to the buccal fat pad during rhytidectomy, in a cadaveric dissection of 19 hemifaces, Hwang et al reported that the parotid duct most commonly crosses superficial to the buccal extension of buccal fat pad (42.1% of dissections), whereas it was found to cross deep to the buccal extension of buccal fat pad in 26.3% of dissections, and along the superior border of the buccal extension of buccal fat pad in 31.6% of specimens.^17^

### Operative Technique for the Intraoral Approach

During the intraoral approach to BFPR, regardless of whether performed under local or general anesthesia, the most critical landmark to identify before making an incision is the papilla of Stensen’s duct, located at the level of the second maxillary molar. Before incision, the author’s preferred technique begins with cleaning the oral cavity with chlorhexidine antiseptic mouthwash. Next, 1% lidocaine mixed with 1:100,000 epinephrine is injected into the gingivobuccal sulcus at the level between the first and second maxillary molar. A 1.5-cm intraoral incision is then made approximately 1 cm inferior to the ampulla of Stenson’s duct. Blunt dissection is carried down through the mucosa and buccinator muscle, while the fascia is spread gently until the fat pad becomes apparent. The fat pad can be exteriorized into the mouth through gentle traction in conjunction with external pressure applied to the cheek and gentle dissection with sterile cotton tip applicators.^2-5^ In avoiding excess traction or pulling, the surgeon can avoid over-resection and injury to the parotid duct or adjacent facial nerve branches. Excessive traction or aggressive dissection will likely result in removal beyond the buccal extension of the buccal fat pad, which may contribute to overly excavated appearance. Electrocautery is used to cauterize the base of the fat pad before resection to ensure that the pedicle and base are cauterized. The typical volume of resected fat is approximately 3-5 g per side but is patient dependent and may vary from each side of the face depending on the preoperative appearance. The incision is closed with absorbable 4-0 chromic sutures, and patients are maintained on a soft diet for approximately 3 days.

## Discussion

### Diagnosis and Clinical Presentation of Parotid Duct Injury

To recognize the clinical appearance of a parotid duct injury, it is crucial to first understand what an expected postoperative appearance should be following BFPR. Patients undergoing BPFR commonly develop mild bilateral facial swelling, which is an expected part of the normal postoperative course and typically resolves within 2-3 weeks. If postoperative swelling persists beyond the typical time frame and hematoma and/or infection have been clinically excluded, the diagnosis of parotid duct injury and/or sialocele should be considered, especially if there is isolated fullness overlying the trajectory of the parotid duct from the papilla to the gland or if the fullness is unilateral (Table 1).

**Table 1.** Clinical Presentation, Signs, and Symptoms of Parotid Duct Injury Following Buccal Fat Pad Reduction

Clinical presentation, signs, and symptoms of parotid duct injury following buccal fat pad reduction
• Continued facial fullness beyond appropriate/expected postoperative edema (>2-3 weeks) • Soft, non-tender, and mobile mass within the parotid region, and possibly also serous drainage from the wound. • Patients may also experience additional swelling or increased drainage with eating sour or spicy food. • Pain overlying parotid due to compression of surrounding tissues.

Given that parotid duct injury may have a subtle presentation, which can initially be misdiagnosed as a hematoma or seroma, performing a thorough history and physical examination often provides useful information in the workup of a suspected parotid injury. Patients with a parotid duct injury commonly present with a soft, non-tender, and mobile mass within the parotid region, and possibly also serous drainage intraorally from the wound. Patients may also experience additional swelling or increased drainage with eating, a symptom that should raise one’s suspicion for a parotid duct injury.

In equivocal cases or when there is a clinical suspicion for parotid duct injury, analysis of aspirated fluid from the mass, if present, can help confirm the presence of a sialocele by demonstrating amylase levels above 100,000 U/L. Additionally, radiographic modalities, such as ultrasound, computed tomography scan, magnetic resonance sialography, or fluoroscopic-guided sialography, can also be valuable in characterizing parotid duct injury if the clinical examination is equivocal.^14,18-21^ Perhaps the most useful radiologic tool helpful in assessing cheek fullness when parotid duct injury is suspected is a sialogram, which involves cannulation of the lumen of the parotid duct, retrograde injection of a contrast medium, and assessment of contrast extravasation from a presumed site of injury.

### Clinical Case Example

**Figure 1.**
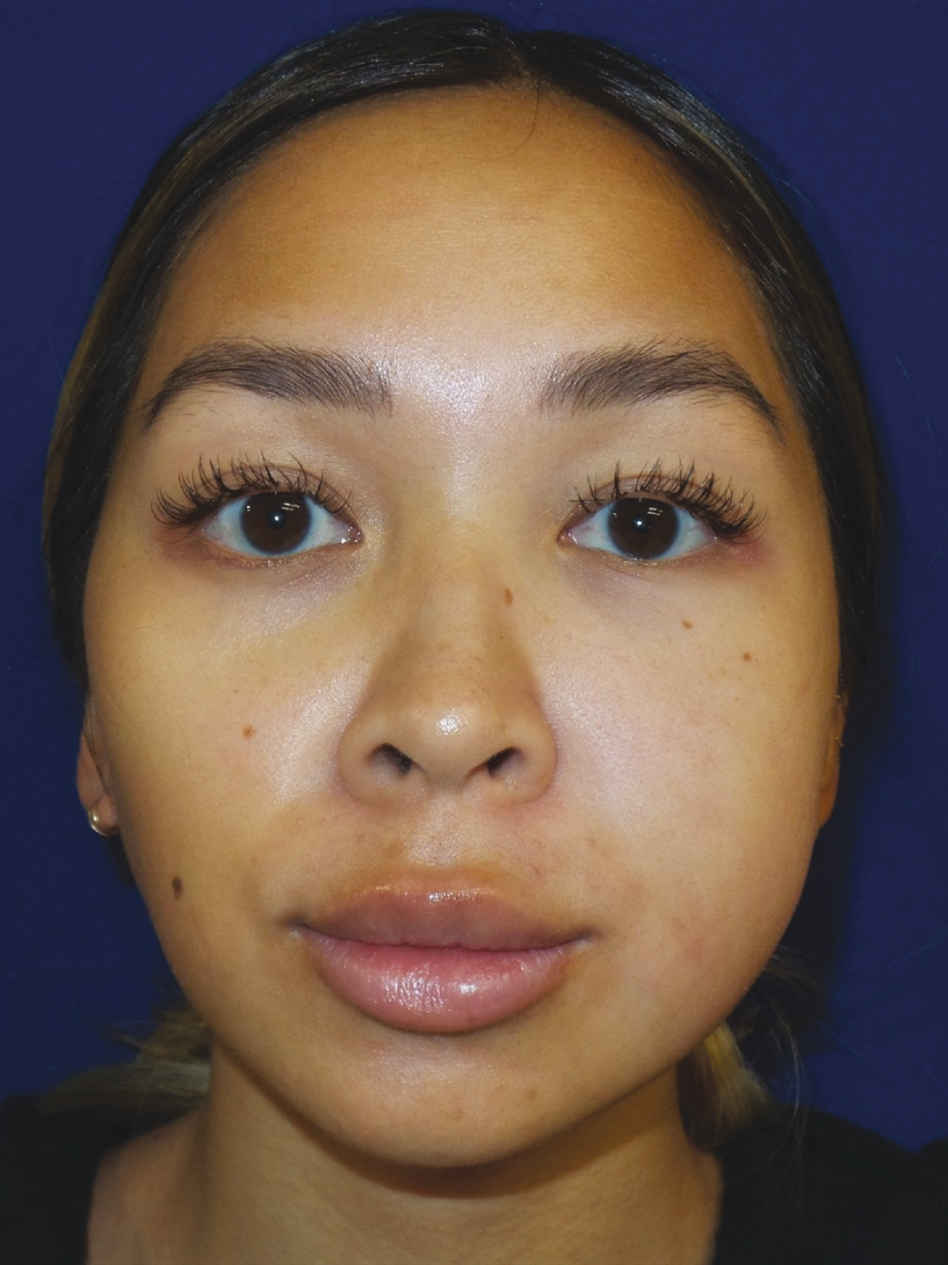
Representative anteroposterior photograph of a 25-year-old female patient who underwent intraoral buccal fat pad removal surgery. This is a 2-week postoperative photograph taken at the time of initial consultation after a referral from another provider. The photograph illustrates the abnormal and asymmetric swelling of the left hemiface.

**Figure 2.**
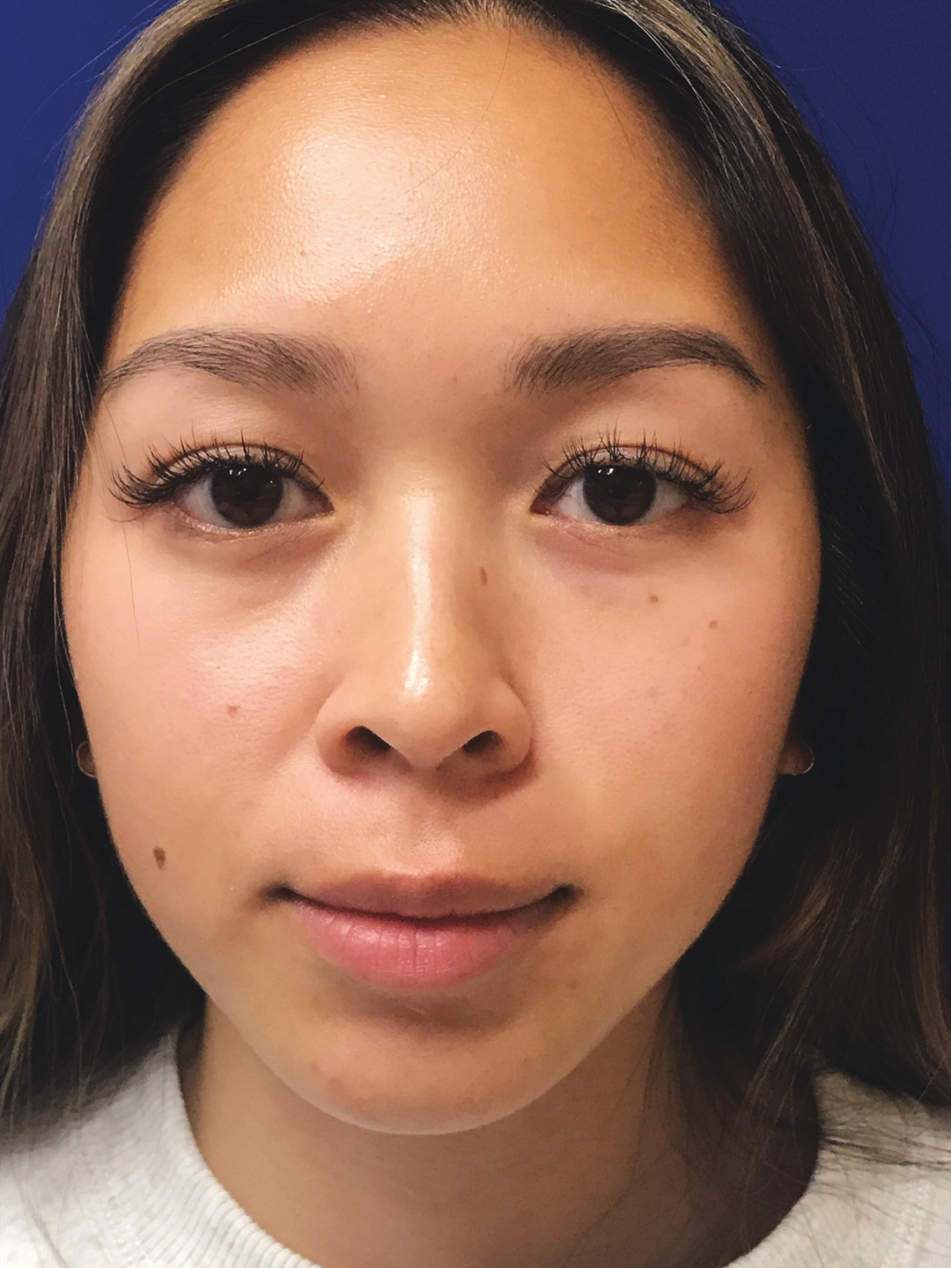
Representative anteroposterior photograph of a 25-year-old female patient 10 days after her first percutaneous aspiration. The photograph was taken after a series of 3 percutaneous aspirations of the left cheek, maintenance in a compression wrap garment, injection of 25 units of Botulinum toxin into the left parotid gland, and initiation of glycopyrronium bromide (Robinul). Following these interventions, the drainage had stopped, and the patient appeared to be healed without further sequelae.

A 25-year-old otherwise healthy female underwent intraoral bilateral BFPR for midfacial contouring with another provider. On the first postoperative day, the patient resumed consumption of a regular diet and immediately noticed increased swelling of the left hemiface. On postoperative day 3, the patient noticed asymmetrically and acutely worsening left cheek fullness. On postoperative day 8, she reported that with manual pressure, she noticed a yellow serous drainage from the intraoral incision on the left side of her mouth. During this time, the patient remained on prophylactic oral antibiotic therapy and remained afebrile. She was referred to our practice for evaluation 2-weeks following BFPR (Figure 1). At this time, the clinical suspicion was that she had sustained a parotid duct injury given her presentation symptoms and examination. Based on our high index of suspicion for a sialocele, no imaging was performed. However, percutaneous aspiration of the left cheek collection was performed yielding 5 mL of serous fluid which was sent for amylase, which ultimately returned higher than 100,000 U/L confirming our presumptive diagnosis. She was placed in a compression wrap garment and was seen back 2 days later for repeat aspiration (3 mL serous fluid). During this visit, a total of 25 units of Botulinum toxin was injected into the left parotid gland, and the patient was started on glycopyrronium bromide (Robinul). The patient then returned for the third aspiration (<1 mL serous fluid). Following these interventions, the drainage had stopped, and the patient appeared to be healed without further sequelae after the 10th day following treatment (Figure 2). Written consent was provided for use of the patient’s photographs.

### Treatment

**Figure 3.**
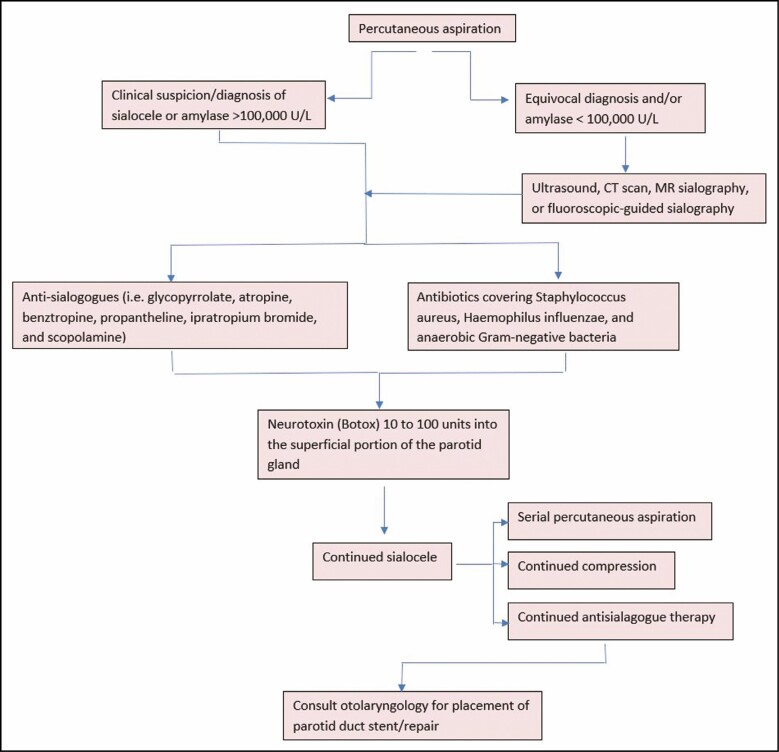
Proposed clinically actionable algorithm for managing a suspected or diagnosed parotid duct injury following intraoral buccal fat pad removal surgery. CT, computed tomography.

Treatment of iatrogenic parotid duct injury is initiated on detection and is managed systematically often in a stepwise fashion, as outlined in Figure 3. As with the management of all complications, it is important to have the patient return to the clinic frequently for serial examinations. Previous publications on parotid duct injuries have described various modalities of treatment, such as serial percutaneous needle aspiration, pressure dressings, antisialagogue therapy, antibiotic therapy, botulinum toxin, and surgical techniques, including duct repair, diversion, ligation, stenting, drain placement, and even parotidectomy. However, there is no consensus regarding the ideal management of these injuries, regardless of the etiology of the injury.^22-25^ With regard to the prevention of bacterial overgrowth, antibiotic coverage should be considered, and antibiotic selection should cover *Staphylococcus aureus*, *Haemophilus influenzae*, and anaerobic Gram-negative bacteria. The antibiotics of choice include the second- and third-generation cephalosporins, penicillins, or clindamycin.^18^

Although the management of parotid duct injury following BFPR has not previously been reported, a previous publication published by Lawson et al discusses the important considerations with regard to the management of iatrogenic parotid duct and parotid parenchymal injuries following facelift surgery.^13^ For parotid salivary leaks following facelift surgery, several applicable treatment strategies have been described and coincide with the aforementioned approaches, including aspiration and drainage strategies, compression, dietary modifications, and pharmacologic modalities.^13^

Once fluid has been percutaneously aspirated as part of the diagnostic evaluation of the fluid and/or the diagnosis of parotid duct injury has been made clinically based on physical examination, the systematic treatment approach should also incorporate other modalities as outlined in Figure 1. First, compression with foam pads and a gentle headwrap can be utilized to provide constant compression to the region of injury. In conjunction with compression, administration of antisialagogue medications can help suppress salivary flow and mitigate the autolysis of the soft tissues, which may contribute to infection or delayed healing. The most commonly used medications to promote these antisecretory effects are anticholinergic drugs, such as glycopyrrolate, atropine, benztropine, propantheline, ipratropium bromide, and scopolamine.^13,24-27^ Despite the beneficial pharmacologic properties of these medications, it is important to educate patients on the potential untoward side effects of some of the oral formulations of these medications, including constipation, dryness of the mouth/nose, blurred vision, difficulty with micturition, drowsiness, headaches, photophobia, nausea/vomiting, and fatigue.^13^ If the side effects of the oral medications are not well tolerated, transdermal scopolamine offers antisialagogue properties with a more limited side-effect profile.

More recently, the use of botulinum toxin has been demonstrated to offer valuable pharmacologic properties in managing parotid leak following either parenchymal injury or isolated parotid duct injury following facelift surgery. Physiologically, it is an anticholinergic agent that acts on the presynaptic receptor by blocking the release of acetylcholine into the neuromuscular presynaptic membrane, causing chemical denervation and a temporary neuromuscular blockade of autonomic cholinergic fibers allowing for suppression of salivary flow. Furthermore, various administration techniques have been described, yet no consensus currently exists regarding the ideal volume or approach to avoid the paralysis of the facial nerve when blindly injecting the neurotoxin around the gland.^13^ Although there appears to be no uniform dosage or technique for administration, some authors have suggested injecting between 10 to 100 units of Botox (Allergan, Irvine, CA) into the superficial portion of the parotid gland using various administration techniques, including ultrasound-guided, under concomitant electromyography, or blindly into the gland.^13^

If the combination of compression, antisialagogues, and neurotoxin injection fails to cease the salivary fluid production with continued evidence of a sialocele, serial percutaneous aspiration should be considered. If these modalities fail to address the salivary production, surgeons with adequate training and comfort may elect to perform repair of the duct over a stent if appropriate or may refer to a colleague in their group or community to assist.

### Management Algorithm

Given the available literature on the management of parotid duct injury whether traumatic or iatrogenic, we believe that the mainstays of managing a parotid duct injury once diagnosed in the setting of BFPR should include (1) early aspiration/decompression and close observation with oral antibiotic therapy, (2) administration of antisialagogue medications, (3) chemodenervation of the parotid gland with botulinum toxin if symptoms persist, and (4) repair and/or stent placement if no resolution has occurred (Figure 1). It should also be noted that, given the intraoral approach, an iatrogenic injury to the parotid duct may go unrecognized during the procedure. Therefore, timely diagnosis and the initiation of appropriate treatment are critical to reduce subsequent complications.

## Conclusions

Intraoral BFPR has become an increasingly common procedure performed to improve facial contour and balance among patients with buccal lipodystrophy or buccal fat pad pseudoherniation. Iatrogenic injury to the parotid duct is an exceedingly rare complication following intraoral buccal fat removal. Given the increasing popularity of this procedure over the recent years through social media platforms, aesthetic surgeons should be familiar with the relevant anatomy of the parotid duct and its relationship to the buccal fat pad. Because there remains a gap in the literature with regard to the management of iatrogenic parotid duct injury in the setting of intraoral BFPR, the authors propose a proper diagnostic approach and treatment algorithm to manage this untoward complication. 

## Notes

Presented at: The Aesthetic Meeting, April 2022, San Diego, CA, USA.
